# Organo-Clay Nanomaterials Based on Halloysite and Cyclodextrin as Carriers for Polyphenolic Compounds

**DOI:** 10.3390/jfb9040061

**Published:** 2018-11-03

**Authors:** Marina Massaro, Serena Riela

**Affiliations:** Dipartimento di Scienze e Tecnologie Biologiche, Chimiche e Farmaceutiche (STEBICEF), University o Palermo, Viale delle Scienze, Ed. 17, 90128 Palermo, Italy; marina.massaro@unipa.it

**Keywords:** halloysite, cyclodextrin, nanosponges, polyphenolic compounds

## Abstract

Hybrid material based on halloysite covalently linked to a hyper-reticulated cyclodextrin network was investigated as a potential carrier for polyphenolic compounds. The absorption ability of the hybrid system was studied in different pH conditions as well as the kinetic release of curcumin, chosen as a drug model. A preliminary study was performed to assess the antioxidant capacity of the obtained carrier. The obtained results highlighted that the curcumin molecule can have sustained release from the carrier over the time, retaining its antioxidant properties due to the combination of two different host systems that give rise to an hyper-reticulated structure, allowing an increase in the drug loading and stabilization. Therefore, this work puts forward an efficient strategy to prepare organic-inorganic hybrids with three different cavities that could encapsulate two or more drug molecules with different physico-chemical properties.

## 1. Introduction

Halloysite nanotubes (HNTs) are an aluminosilicate clay with a predominantly hollow tubular structure and chemically similar to the platy kaolinite (Al_2_Si_2_O_5_(OH)_4_). Being a natural materials, HNT is available at low cost and it has been shown to have excellent bio- [1] and eco-compatibility [2]. Generally, the inner and outer diameters of the tubes are in the ranges of 10–30 nm and 40–70 nm, respectively, while their length is in the range of 0.2–1.5 μm [3]. The halloysite tubes are positive in the inner lumen, where are located mostly of the aluminum hydroxide, whereas the external surface is negative, due to the presence of silicon dioxide [4]. Due to these features, halloysite nanotubes can be selectively functionalized at the outer and/or inner surfaces leading to the synthesis of several interesting nanomaterials [5,6]. HNTs are indeed widely used as drug carrier [7,8,9,10,11] and delivery systems [12], enzyme immobilization [13], pollutant removal [14,15,16,17], catalyst [18,19,20], and so on.

Cyclodextrins (CDs), formed by six (α-CD), seven (β-CD), or eight (γ-CD) α-(D)-glucopyranose units, are very promising materials for their ability to act as molecular [21,22] and chiral selector [23]. Studies on CDs are the object of an immense literature that is periodically reviewed [24,25]. In this context, different studies have shown that their complexation ability with, for example, some α-amino acids or small peptides could be dependent on the presence of substituents on the primary or secondary rim [23]. The complexation processes that are involved, between CD and guest molecules, are highly important in several fields especially regarding the development of drug delivery systems.

In the last years, organo-clay hybrid nanomaterials based on halloysite covalently linked to modified-cyclodextrin units were reported as carriers for polyphenolic compounds. These nanomaterials showed simultaneously carrier ability for different natural drugs [26,27]. 

Recently, nanosponges [28,29], obtained by combining supramolecular hosts such as cyclodextrins, have attracted great attention since they allow one to increase the loading ratio of biological active molecules into the carrier [30]. 

**Figure 1:**
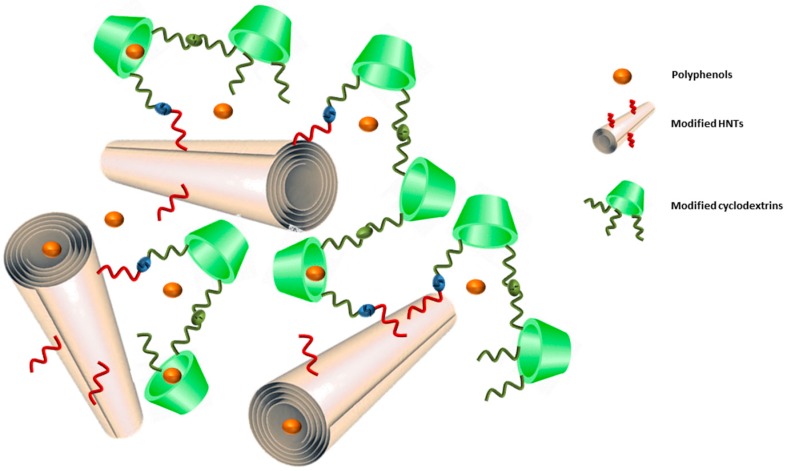
Schematic representation of the HNT-cyclodextrin (HNT-CD) hyper-reticulated hybrid.

Herein, we present a preliminary investigation on the development a potential drug carrier system for biologically active compounds, based on halloysite functionalized with a hyper-reticulated cyclodextrin network (HNT-CD) (Figure 1). The HNT-CD hybrid, which possesses “three” different cavities, namely the HNT lumen, the β-CD, and additional nanochannels between the cross-linked hosts, could lead to an increase in the loading of drugs with respect to the non-reticulated one. Furthermore, it could offer the remarkable possibility of achieving a controlled and sustained release of one or more drugs in agreement with the interacting cavity.

## 2. Results and Discussion

**Scheme 1:**
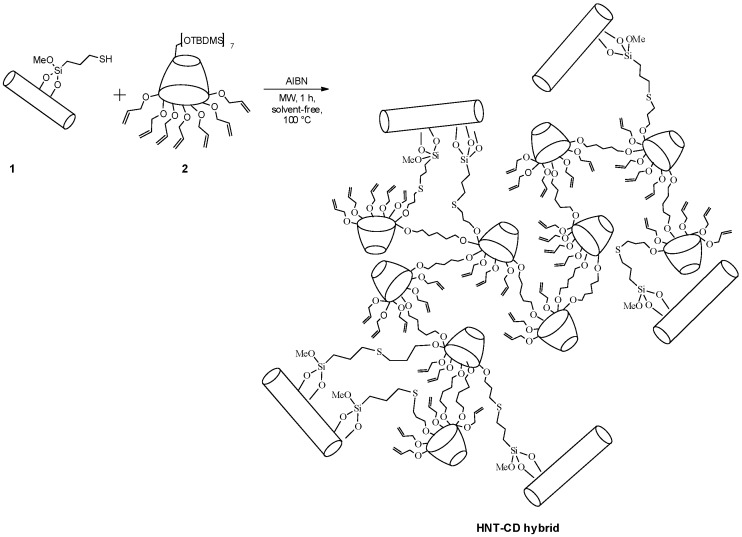
Synthetic route for the synthesis of HNT-CD hybrid.

The synthesis of the organic–inorganic hybrid was accomplished, by a one step thiol-ene reaction, as reported in Scheme 1. In particular, modified halloysite, possessing a degree of functionalization of 0.2 mmol·g^−1^ in thiol groups, was mixed with heptakis-6-(tert-butyldi-methylsilyl)-2-allyloxy-β-cyclodextrin in a 1:1.5 HNT:CD ratio. In this way, a hybrid with 40 wt % CD covalently grafted on halloysite external surface was obtained [31]. The reaction was performed by microwave irradiation and in solvent-free conditions. The HNT-CDs hybrid showed a surface area of 19.9 m^2^·g^−1^, slightly smaller than that of starting pristine halloysite (22.1 m^2^·g^−1^). Furthermore, the physico-chemical characterization of this nanomaterial showed the presence of a hyper-reticulated structure with different “cavities”; i.e., the halloysite lumen, the β-CD cavity and some nanochannels due to the crosslinking through the hybrid structure. Furthermore, swelling studies showed that the hybrid possesses a swelling ratio of 91.2 ± 1.6% [31]. All these features are crucial in determining the interactions of the hybrid with guest molecules, so they promote the synthesized nanomaterial as a potential efficient drug carrier system for one or more different drugs. Preliminary investigations were performed choosing as a drug model three different polyphenolic compounds, i.e., curcumin, silibinin, and quercetin.

Previous investigations have shown that native β-CD and the modified one can positively interact with both quercetin and curcumin, forming 1:1 and 1:2 supramolecular complexes [26,27]. Furthermore, the two molecules can also interact with the HNT lumen by means of electrostatic interactions and hydrogen bonding. Conversely, fluorescence and UV-vis studies have shown that silibinin does not interact with the β-CD cavity, but only with the HNT internal surface [26].

Based on these findings a preliminary study was performed in order to evaluate the binding abilities of a hyper-reticulated HNT-CD hybrid toward quercetin, curcumin, and silibinin.

The adsorption capacity of the hybrid for the selected biological molecules was evaluated under two different pH values, namely, 1.0 and 6.0. These values were chosen according to the pK_a_ values of the drugs (for quercetin, pK_a1_ = 7, pK_a2_ = 9, and pK_a3_ = 11; for curcumin, pK_a1_ = 8.38, pK_a2_ = 9.88, and pK_a3_ = 10.51; for silibinin, pK_a1_ = 7.87 and pK_a2_ = 9.57, respectively), so it was possible to study the interaction with the HNT hybrid in the anionic, neutral, or cationic form of the molecules. 

**Figure 2:**
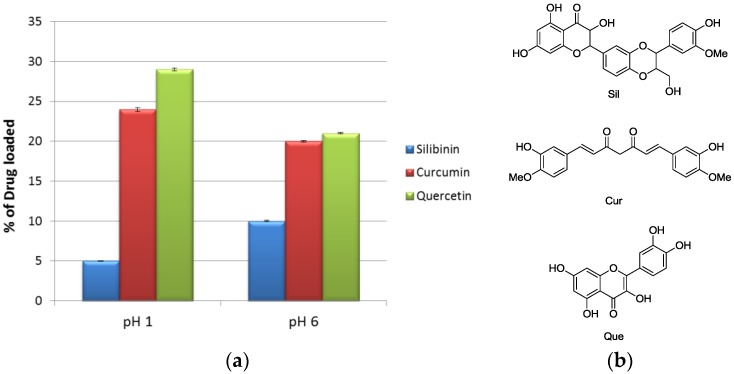
(**a**) Amount of quercetin, silibinin, and curcumin loaded in the HNT-CD hybrid; (**b**) The structure of the investigated polyphenols. Relative errors are in a range of ±0.5–1% (results correspond to the mean ± standard error mean of three independent assays).

The obtained results, calculated by using Equation (1), are shown in Figure 2.

As can be easily seen, the hybrid can absorb quercetin and curcumin in both the pH investigated, conversely silibinin is barely absorbed both at pH 1 and pH 6. In the last case, a slight increase in the absorption rate of the HNT-CD hybrid toward silibinin is observed. These results can be explained as follows. In acidic solution, where the silibinin molecule is positively charged as well as the halloysite lumen, electrostatic repulsions are present, and the adsorption capacity of the system is therefore limited. By increasing the pH, the silibinin molecule could be partially dissociated, so it is less soluble in aqueous medium and can therefore be loaded into HNT as a consequence of hydrophobic effects.

As for quercetin, its adsorption ratio onto the HNT-CD hybrid reflects the retention efficiency of Que on β-CD, which depends on the pH of the medium [26]. It is, indeed, reported that, in acidic solution, strong interactions exist between quercetin and the CD cavity.

On the basis of the above findings, a solid complex between the obtained hybrid and curcumin (chosen as drug model) was synthesized.

The loading of curcumin into the HNT-CD hybrid was carried out by mixing the hybrid compound with a methanolic highly concentrated curcumin solution following a procedure reported elsewhere [32]. After loading, the HNT-CD/Cur complex was washed with water to remove free drug. Cur-loaded pristine HNT, HNT/Cur, was similarly prepared as a reference material. The drug loading of HNT/Cur and HNT-CD/Cur was estimated by UV-vis measurements. The amounts of Cur loaded in the HNT nanomaterials, expressed as the percent amount of drug in the final composite, were ca. 1.5 wt % and 7.7 wt % for HNT/Cur and HNT-CD/Cur, respectively, with encapsulation efficiencies of 41% and 94% for pristine HNT and HNT-CD hybrid, respectively (Equations (2) and (3)).

The different percent loadings of drug in the different carriers can be explained, as already reported for similar carrier systems [32], by the specific functionalization of HNT external surface that promotes the curcumin adsorption because of the introduction of CD moieties and the formation of a polymeric network, so the drug can be efficiently loaded on three different cavities, the HNT lumen, the CD one, and the channels between the cross-linked CD and HNT units.

To evaluate the performances of the HNT-CD/Cur hybrid complex for application in the drug carrier field, preliminary investigations were performed; in particular, the kinetic release of the drug from the hybrid system and the antioxidant properties of the organic–inorganic complex were evaluated.

**Figure 3:**
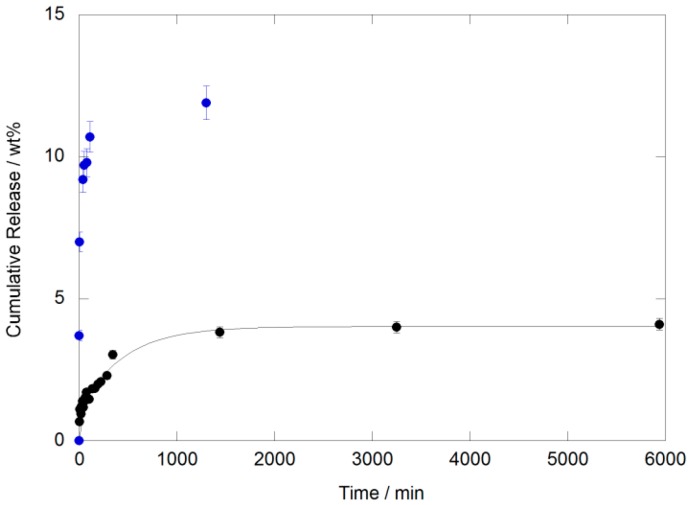
Kinetic release of curcumin from HNT-CD hybrid nanomaterials (blue line non-hyper reticulated CD covalently linked on halloysite and black line HNT-CD hybrid) in a phosphate buffer pH 7.4.

The release of curcumin loaded in HNT-CD hybrid was evaluated by the dialysis bag method using conditions designed to mimic physiological conditions (phosphate buffer pH 7.4) and the obtained kinetic data (Equation (4)) are shown in Figure 3. As it is possible to note, in these conditions the HNT-CD hybrid retains almost the total amount of the loaded curcumin for at least 96 h. These results are very different from the curcumin kinetic release from a carrier system where to HNT, is covalently grafted a single cyclodextrin unit [33], in this latter case indeed, about 15 wt % of curcumin was released after 24 h (Figure 3), which followed an initial burst release. Therefore, the introduction of a hyper-reticulated cyclodextrin core onto the HNT surface was crucial to slow down the drug release in physiological conditions, obtaining a sustained release of the biological molecule within time.

The kinetic data obtained were analyzed by several models to deep investigate the release behavior of curcumin from such a complex system (Table 1). The results showed that the data are better fitted by a double exponential model (DEM) (*R*^2^ = 0.9824) with *k*_1_ = 0.2 ± 0.1 min^−1^ and *k*_2_ = 0.0022 ± 0.0003 min^−1^. According to the literature, the DEM describes a mechanism consisting of two parallel reactions involving two distinguishable species [34]. Therefore, in our case, we observed the release of curcumin from both the cyclodextrin cavity and the HNT lumen.

As reported in literature, the curcumin molecule possesses an intrinsic antioxidant activity due to its peculiar chemical structure. Due to this activity, curcumin has been shown to inhibit lipid peroxidation and to manifest free radical-scavenging activity, in particular toward oxygen and nitrogen-centered free radicals [35]. To evaluate if the molecule retains its antioxidant ability once encapsulated into the HNT-CD hybrid system, we assessed the radical trapping ability of the HNT-CD/Cur complex by studying the reaction with 2,2-diphenyl-1-picrylhydrazyl radical (DPPH). 

**Figure 4:**
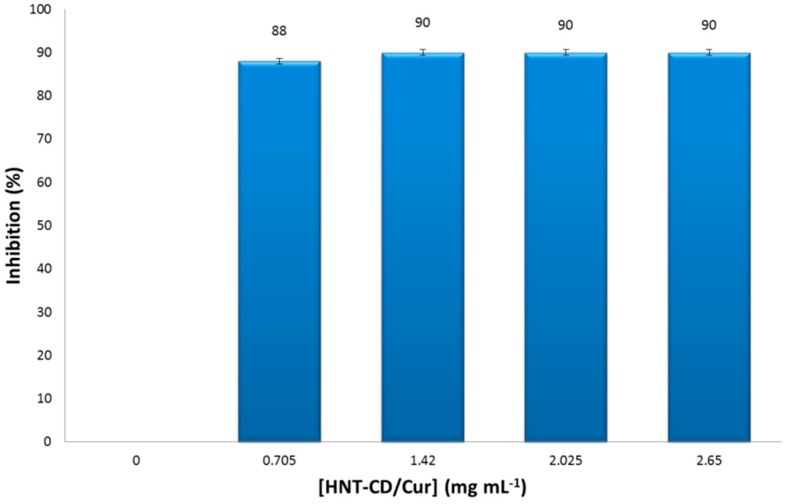
Antioxidant activity of the HNT-CD/Cur complex. Relative errors are in a range of ±0.5–1% (results correspond to the mean ± standard error mean of three independent assays).

The antioxidant capacity of the HNT-CD/Cur complex in MeOH was evaluated by plotting the percentage of inhibition of the free radical scavenging capacity of DPPH (Equation (5)) against the concentrations of different dispersions of HNT-CD/Cur (from 0.7 to 2.56 mg·mL^−1^) (Figure 4). 

The purple DPPH solution quickly faded to yellow with the addition of a small amount of the HNT-CD/Cur complex indicating an excellent antioxidant activity, of the overall system, with an IC_50_ lower than 0.7 mg·mL^−1^ (Figure 4). In the same experimental condition, pristine HNT did not exhibit any radical scavenging activity [36]. Therefore, the curcumin molecule loaded into the HNT-CD hybrid system preserves its antioxidant properties.

Undoubtedly, the results presented herein have gone one step further for curcumin delivery for the development of efficient carrier as alternative route for the drug administration to the already reported systems. Nanosponges for the delivery of curcumin have been already reported, but these systems do not ensure the sustained release that we have reached by combining the properties of halloysite and β-CD [37,38]. Furthermore, by comparing our preliminary results with the ones obtained using other hybrid systems for the delivery of curcumin [39], one can conclude, in a first attempt, that the as-prepared hyper-reticulated network could effectively improve the stability and release profile of curcumin in simulated physiological conditions, indicating their promising potential for oral delivery.

## 3. Materials and Methods 

HNT-CD hybrid was synthesized as reported elsewhere [31]. 

UV-vis measurements were performed using a Beckmann DU 650 spectrometer (Beckmann Instruments, Inc., Fullerton, CA, USA).

The specific surface area and pore site distribution was made by N_2_ adsorption/desorption Nova 2200 Quantachrome Instruments analyzer (Quantachrome Instruments, Boynton Beach, FL, USA).

### 3.1. Synthesis of the HNT-CD Hybrid

Thiol-functionalized HNTs (200 mg) and heptakis-6-(tert-butyldimethylsilyl) 2-allyloxy-β-cyclodextrin (300 mg) were weighed in an MW test tube provided with a cap and a catalytic amount of AIBN was added. The mixture was inserted in the MW apparatus at 100 °C, under constant stirring, for 1 h, with an initial set power of 80 W. Successively, the solid was filtered off, rinsed several times with CH_2_Cl_2_ and CH_3_OH (until the unreacted reagents were not detected by TLC), and dried at 80 °C under vacuum.

### 3.2. Batch Adsorption Experiments

All adsorption experiments were conducted on sealed vessels containing 2.5 mg of HNT-CD hybrid and 1 mL of drug solution (2 × 10^−5^ M) in HCl 0.1 N and phosphate buffer pH 6.0 at 298 K, respectively. The obtained dispersions were vigorously vortexed and shaken in a thermostatic shaker with a shaking of 200 rpm at 298 K overnight. The supernatants were obtained by centrifugation for detecting drug concentration via UV-vis spectrophotometer at the maximum absorption wavelength of each drug (280, 420, and 360 nm for silibinin, curcumin, and quercetin, respectively).

The loading efficiency (*LE*) was estimated by using the following equation:

(1) $$LE(\%)=\frac{\left(C_{0}-C_{e}\right)}{C_{0}}\times 100$$

where *C*_0_ and *C*_e_ are initial and equilibrium concentrations of the drugs (M), respectively. 

### 3.3. Loading of Curcumin in the HNT-CD Hybrid

To a dispersion of HNT-CD hybrid (100 mg) in water (5 mL), 1 mL of a solution 10^−2^ M of curcumin in MeOH was added. The suspension was sonicated for 5 min, at an ultrasound power of 200 W and at 25 °C and then was evacuated for 3 cycles. The suspension was left under stirring for 24 h at room temperature. After this time, the powder was washed with water and then dried at 40 °C.

The amount of Cur in the supernatant solution was assessed by UV-vis investigations.

The drug loading and encapsulation efficiencies (*LD*% and *EE*%, respectively) were calculated according to the following equations:

(2) $$LD\%=\frac{Cur_{HNT}}{m_{HNT-CD}+Cur_{HNT}}\times 100$$

(3) $$EE\%=\frac{Cur_{HNT}}{Cur_{TOT}}\times 100$$

where *Cur_HNT-CD_* and *Cur_TOT_* are the amount of Cur loaded into HNT nanomaterials and the total feed Cur, respectively, and *m_HNT-CD_* is the amount of HNT-CD hybrid.

### 3.4. Drug Release Study

The release of Cur from the HNT-CD hybrid was done as follows: 20 mg of the sample were dispersed in 1 mL of dissolution medium and transferred into a dialysis membrane (Medicell International Ltd., London, UK; MWCO 12-14000 with a diameter of 21.5 mm). Phosphate buffer (0.05 M, pH 7.4, (fresh prepared)) was used as the release medium. Subsequently, the membrane was put in a round bottom flask containing 10 mL of the release medium at 37 °C and stirred. At fixed time, 1 mL of the release medium has been withdrawn and analyzed by UV-vis measurements. To keep constant the volume of the release medium, 1 mL of fresh solution has been used to replace the collected one. Total amounts of drug released (*F_t_*) were calculated as follows:

(4) $$F_{t}=V_{m}C_{t}+\sum_{i=0}^{t-1}V_{a}C_{i}$$

where *V_m_* and *C_t_* are the volume and the concentration of the drug at time *t*. *V_a_* is the volume of the sample withdrawn, and *C_i_* is the drug concentration at time *i* (*i* < *t*). 

To determine the release mechanism, the amount of released molecules vs. time was studied using the first-order, Korsemeyer-Peppas, Higuchi, Hixon, and Crowell and the double exponential (DEM) mathematical models where *M_t_* is the drug release fraction at time *t*, *M*_0_ is the initial amount of drug in the HNT-CD hybrid, *M_HNT-CD_* is the amount of drug remaining into HNT-CD at time *t*, *k* is the kinetic release constant of the respective equations, *t* is the release time, and *n* is the characteristic diffusion exponent, depending on the release mechanism and the geometry of device. If Fickian diffusion occurred, n decreased to 0.5 for slab/cylinder/sphere. If non-Fickian (anomalous) diffusion dominated, the *n* value was between the above value corresponding to the polymer chain relaxation (*n* < 1) for slab/cylinder/sphere. Thus, through determination of the *n* value, the drugs release mechanism could be identified.

### 3.5. Antioxidant Activity

The antioxidant properties were evaluated using the 2,2-diphenyl-1-picrylhydrazyl radical (DPPH•) method. This radical is commonly used to evaluate the antioxidant properties of natural products, as the purple free radical is transformed by reductants to the yellow hydrazine through a formal hydrogen atom transfer reaction. The interaction of HNT-CD/Cur with DPPH radicals resulted in fast decoloration of the DPPH solution. Radical scavenging activity was determined following as follows. The HNT-CD/Cur hybrid (concentration ranging from 0.7 to 2.65 mg·mL^−1^) was dispersed in 1 mL of MeOH. One milliliter of DPPH solution (1.5 × 10^−4^ M) in MeOH was then added to the tested sample. The mixture was then vigorously mixed and allowed to stand at room temperature in the dark for 60 min. The inhibition of free radical DPPH in percent (*I*%) was calculated as:

(5) $$I\%=\left(\frac{A_{c}-A_{HNT-CD/Cur}}{A_{c}}\right)\times 100$$

where *A_c_* is the absorbance of the DDPH solution without HNT-CD/Cur (λ = 523 nm), and *A_HNT-CD/Cur_* is the absorbance of the test sample. All tests were carried out in duplicate, and inhibition percentages were reported as mean ± SD. 

## 4. Conclusions

We investigated the ability of an inorganic–organic hybrid based on halloysite and hyper-reticulated cyclodextrin as a carrier for polyphenolic compounds. Preliminary studies highlighted that the carrier is promising for the delivery of quercetin and curcumin and, in the last case, can act as reservoir for the prolonged release of the drug over 96 h. Antioxidant measurements showed that the curcumin in the hybrid retains its properties, which could be beneficial for the treatment of several pathologies. Current work is devoted to assessing the feasibility of the hybrid system antioxidant/antitumoral agent by means of in vitro and in vivo studies. In this context, the hybrid system developed, still showing unreacted groups available for further functionalization, could be a promising agent for the delivery of polyphenolic compounds by improving their cellular uptake and consequently their therapeutic efficacy.

## Figures and Tables

**Table 1:** Kinetic parameters for the release of curcumin from HNT-CD hybrid in phosphate buffer pH 7.4.

First Order $M_{t}=M_{\propto }\cdot \left(1-e^{-kt}\right)$			Korsemeyer Peppas $M_{t}=k\cdot t^{n}$			Hixon and Crowell $\sqrt[{3}]{M_{0}}-\sqrt[{3}]{M_{HNT-CD}}=k\cdot t$		Higuchi $M_{t}=k\cdot t^{1/2}$		DEM $M_{t}=M^{\prime }\cdot \left(1-e^{-k^{\prime }\cdot t}\right)+M^{\prime \prime }\cdot \left(1-e^{-k^{\prime \prime }\cdot t}\right)$				
*M_∞_* (wt %)	*k* (min^−1^)	*R* ^2^	*k* (min^−1^)	*n*	*R* ^2^	*k* (min^−1^)	*R* ^2^	*k* (min^−1^)	*R* ^2^	*M′* (wt %)	*k′* (min^−1^)	*M″* (wt %)	*k″* (min^−1^)	*R* ^2^
3.8 ± 0.3	0.005 ± 0.001	0.7825	0.58 ± 0.05	0.23 ± 0.01	0.9441	(4.9 ± 0.3) × 10^−8^	0.8379	n.a.		1.01 ± 0.08	0.2 ± 0.1	3.0 ± 0.1	0.0022 ± 0.0003	0.9824
